# The challenges of psychopharmacological treatment during the COVID-19 pandemic in lombardy

**DOI:** 10.1192/j.eurpsy.2021.62

**Published:** 2021-08-13

**Authors:** A. Vita

**Affiliations:** Department Of Clinical And Experimental Sciences University Of Brescia, And Department Of Mental Health And Addiction Services, Spedali Civili Hospital, University of Brescia, Brescia, Italy

**Keywords:** Mental disorders, Psychopharmacology, COVID19

## Abstract

**Introduction:** Public Mental Health Services in Lombardy (Italy) has 27 Departments for Mental Health and Addiction Services and a number of private residential facilities. With the reorganization of the entire Healthcare system to deal with COVID-19, Regional Health Authorities recognized mental health as a priority and authorized the continuation of mental health services for the general population. **Objectives:** To review the initiatives and procedures implemented in Lombardy during the Covid-19 pandemic in relation to the organization of Psychiatric Services and continuity of psychopharmacological treatment. **Results:** Hospital admissions for acute psychiatric disorders in patients positive for COVID-19 required a dedicated area in the psychiatric ward or alternatively, a medical ward supported by psychiatric staff. Psychiatric hospital activity for patients negative for Covid-19 has been maintained as usual. The activity in the Mental Health Centers has been maintained in patients suffering from severe mental disorders as well as in those with serious social problems or judicial sentences. Particular attention was paid to patients’ clinical monitoring and drug administration. Long-acting Injection antipsychotics were often preferred to oral treatment to ensure adherence and continuity of care. Appropriate e-health technologies were used to reach patients and their families, for monitoring patients and avoiding drop-outs of patients with serious diseases. **Conclusions:** Maintaining continuous monitoring of patients in contact with mental health services is essential for a careful assessment of their condition from both a psychopathological and medical point of view during pandemic.

**Disclosure:**

No significant relationships.

